# Early acute cerebellar ataxia after meningococcal B vaccine: a case report of a 7-month-old infant and a review of the literature

**DOI:** 10.1186/s13052-023-01480-1

**Published:** 2023-06-05

**Authors:** Nicola Adriano Monzani, Antonio Corsello, Claudia Tagliabue, Raffaella Pinzani, Eleonora Mauri, Carlo Agostoni, Gregorio Paolo Milani, Robertino Dilena

**Affiliations:** 1 Department of Clinical Science and Community Health, University of Milan, Milan, Italy; 2 Fondazione IRCCS Ca’ Granda Ospedale Maggiore Policlinico, Neonatal Intensive Care Unit, Milan, Italy; 3 Fondazione IRCCS Ca’ Granda Ospedale Maggiore Policlinico, SC Pediatria Pneumoinfettivologia, Milan, Italy; 4 Fondazione IRCCS Ca’ Granda Ospedale Maggiore Policlinico, S.C. Neurofisiopatologia, Milan, Italy; 5 Fondazione IRCCS Ca’ Granda Ospedale Maggiore Policlinico, Pediatric Unit, Via Francesco Sforza 35, 20122 Milan, Italy

## Abstract

Acute cerebellar ataxia (ACA) and acute cerebellitis represent disorders characterized by a para-infectious, post-infectious, or post-vaccination cerebellar inflammation. They are relatively common neurologic disorders among children, and may follow infections, or, more rarely, vaccinations. Few cases are instead described among infants. Although the immunization with meningococcal group B (MenB) vaccine has been associated with some neurological side effects, suspected ACA has been reported only once in the literature. Case presentation: we describe a 7-month-old female that presented ACA within 24 h from the MenB second dose vaccination. Extensive laboratory studies and magnetic resonance imaging excluded other causes. We then conducted an extended review of other vaccine related cases reported in the literature, focusing on the clinical characteristics of ACA and finding that ataxia and cerebellitis of para- or post-infectious cause are very rarely described in the first year of life. We collected 20 articles published in the last 30 years, including an amount of 1663 patients (1–24 years) with ACA. Conclusions: a very small number of suspected post-vaccinal ataxias has been described in recent years, compared to other causes, and vaccination remains an unquestionable medical need. Further research is needed to clarify the complex pathogenesis of this disorder and its eventual link with vaccinations.

## Background

Ataxia can be considered a non-specific sign of various disorders that may involve different levels of the central nervous system (CNS), resulting in impaired movements, coordination and balance [1]. Most involved causes are generally represented by damages or inflammation in basal ganglia, cerebellum or cerebral cortex [2]. Childhood ataxia is generally classified by the determination of its clinical course and the characteristics of associated neurological disorders [3]. Childhood ataxia may be both congenital and acquired, and it is usually associated with CNS congenital anomalies, even if its pathogenesis is not yet fully understood [1]. Acute cerebellar ataxia (ACA) represents the most common presentation of childhood ataxia with an estimated incidence of 1:100,000–500,000 children per year [4, 5].

ACA more frequently occurs in early childhood (2–4 years), and it may be observed in older children and adolescents [6]. Cases under 12 months of age are poorly described, representing nearly 2.5% of pediatric patients [7]. The neurological ongoing development and the lack of evident symptoms with consequent reduced hospital access could have a role in the underestimation of this condition in infants and toddlers [8, 9]. ACA is considered an autoimmune response triggered by an infection or, less frequently, a vaccine, with a subsequent antibody cross-reactivity against cerebellar self-antigens [10]. Usually, ACA follows a benign viral infection, and varicella represents the most common one [11]. Other possibly related causes are Epstein Barr virus, enterovirus, hepatitis A, mumps, and Influenza A/B viruses [12–14].

ACA is usually characterized by hypotonia, gait disorders, speech abnormalities, dysmetria, nystagmus and intention tremor [15]. The main concern at onset is to exclude organic causes of acute ataxia, such as CNS infections, strokes, tumors, or intoxications [16]. Some studies published in the late twentieth century reported cases of ACA following vaccinations for varicella, influenza, hepatitis B, meningococcal group C, diphtheria-pertussis-tetanus, papillomavirus [17–21]. In these reports, five females and one male were included. The mean age was 9.9 years, and the latency time between vaccine and onset of infection ranged from 5 h to 12 days [21, 22]. Moreover, the few patients under 2 years-old presented a short-latency period of 5 h from diphtheria-pertussis-tetanus vaccination [19, 22]. Immunization with MenB vaccines has been associated with rare neurological side effects including drowsiness, abnormal crying, headache, convulsions, hypotonia and mild meningitis, but cerebellar symptoms have not been described so far [23–25].

Hereby, we report a case of a 7-months-old female with ACA following MenB vaccination with full recovery within a few days.

## Case presentation

In January 2021, a previously healthy 7-months-old female was admitted to the pediatric emergency room for a poor sitting balance, which was completely achieved 3 weeks before, and abnormal involuntary and oscillatory movements spread to head, limbs and torso. Posture while sitting unsupported was very difficult to be maintained, with oscillations and falls. Symptoms had started about 48 h earlier. A contemporary history of fever and vomit was also referred, resolved at the moment of the admission to the hospital. Parents reported that she had received the second dose of MenB vaccination (Bexsero-Novartis Vaccines and Diagnostics S.r.l., Siena, Italy) 72 h before the admission to the emergency room.

The patient's prenatal and perinatal history was unremarkable, with a normal pregnancy and good adaptation at birth. The patient was born via spontaneous gestation at 39 weeks gestational age, with a birth weight of 3020 g. She was full-term born by vaginal delivery with a 1-min Apgar score of 9 and an adequate birth weight for gestational age. Neonatal screening for congenital hypothyroidism, phenylketonuria, cystic fibrosis, and 17-hydroxyprogesterone resulted negative. The patient was exclusively breastfed at discharge, which occurred after monitoring for an episode of cyanosis on the second day of life. The patient's vital parameters were within the norm, and echocardiography was normal. A blood count at birth revealed mild thrombocytopenia, which was resolved on a follow-up control. The patient was discharged in good general condition with well-established breastfeeding. Developmental milestones were regularly achieved.

According to the patient's medical records, the patient had received the first two doses of the hexavalent vaccine (DTaP-Hib-IPV-HepB) and the first dose of MenB vaccine (Bexsero) without any reported side effects. The hexavalent vaccine was administered at the third and fifth months of life, while the MenB vaccine was given at the fifth month.

At the examination she was awake and manifested neither nystagmus or saccadic eye movements, and her optic fundi and cranial nerves were normal, as her tendon reflexes. In order to exclude an infectious meningoencephalitis, blood sample, CT scan, spinal tap and basal electroencephalogram were performed. The CT scan and the electroencephalogram did not reveal abnormal findings. Upon admission to the emergency room, a thorough diagnostic workup was initiated to identify all the potential causes of ACA in our patient. Blood chemistry, leukocyte count, acute phase proteins, and electrolytes were in normal range. Blood serologies for adenovirus, cytomegalovirus, Epstein-Barr virus, coxsackie virus, herpes simplex virus, human herpesvirus 6, Mycoplasma pneumoniae, parvovirus B19 and came back negative. Cerebrospinal fluid (CSF) analysis found a white blood count of 1/μL, protein level 17 mg/dL (n.v. 10–45 mg/dL), glucose level of 55 mg/dL (n.v. 40–70 mg/dL), lactate 1.9 mmol/L (n.v. 1.1–2.8 mmol/L), with no microscopic evidence of contamination. The workup included a polymerase chain reaction (PCR) analysis of both blood and CSF for a range of viral and bacterial pathogens that are known to cause meningitis and encephalitis. Specifically, we conducted PCR testing for all viruses and bacteria that are considered probable causes of these infections, as well as for mycoplasma pneumoniae and fungal infections. Both blood and CSF cultures were performed at the time of admission to the emergency room but did not yield any positive results.

To further investigate the potential viral etiology of ACA in our patient, we performed additional PCR testing for other viruses that may be associated with this condition. Specifically, we looked for Influenza (A H1, A H3, B) and Parainfluenza viruses, as well as other respiratory viruses such as SARS-CoV-2, rhinovirus, enterovirus, RSV, and adenovirus. Despite these efforts, the molecular and serological search for viral causes of ACA on nasopharyngeal aspirate, CSF and blood did not yield any positive results.

The electrocardiogram found normal sinus rhythm and repolarization. A magnetic resonance imaging of the brain and spinal cord were then performed, with no abnormal findings. A possible correlation between the clinical picture of ACA and the recent vaccination was then suspected as the first diagnostic hypothesis.

To exclude any possible correlation with a hypothetical paraneoplastic etiology, a chest X-array and a complete abdominal ultrasound were performed. Homovanillic and vanilmandelic acid urinary excretion did show any anomalies. The search for specific CSF and blood autoantibodies (anti-glutamic acid decarboxylase, anti-HU, anti-YO and anti-RI) did not find abnormal results. Plasma level of neuron specific enolase was first 21.3 ng/mL (n.v. < 12.5 ng/mL), but then decreased back off to range in the following days.

During the hospitalization her general conditions remained stable and started to improve. The neurological follow up during the hospital stay confirmed the positive evolution with an increased head and trunk control and seated posture maintenance with minimal sway. Acute phase proteins remained in normal range.

Therefore, in view of the temporal correlation between the second dose of MenB vaccine and the onset of symptoms, a pharmacovigilance report was sent. Symptoms gradually improved, and the patient was discharged after an 8-days recovery. Clinical full recovery was confirmed at the 10-days and 1-month follow-up, when she was completely able to maintain a stable seated position with no swinging movements and dysmetria.

## Discussion and conclusions

The peculiarities of this case are represented by the occurrence of ACA of unknown origin after MenB vaccine booster and the unusual very young age of ACA presentation (7 months of age).

We searched on PubMed all the literature of the past 30 years published on “Acute Cerebellar Ataxia” and then identified the articles describing pediatric cases of ACA (Table 1) with the aim to evaluate the cases that could have relationship with vaccination and the cases with young age at presentation (< 12 months). A total of 20 articles have been evaluated, mostly regarding children and adolescents between 1 and 24 years (mean age 4,2 years, males 54.6%), including 1663 patients [5–7, 9, 14, 15, 26–39]. Three articles declared to include infants, but only one study specified the exact number of patients under 1 year old (3 patients out of 120, 2.5%) [7]. The majority of patients included in this study (98/120; 82%) were 1 to 6 years old, while only 19 (16%) were older. Although post-infective is overall the most common cause of ACA, none of the three infants < 1 year of age with ACA had an acute post-infectious cerebellar ataxia. Following the available data on age distribution and respecting the statistical analysis conducted, infants constitute 0.3–2.4% of the total, confirming infrequent reports of ACA in the first year of life.

**Table 1:** Pediatric cases of acute cerebellar ataxia described in recent literature

	*Number of patients*	*Males (%)*	*Females ****(****%)*	*Age range (years)*	*Median age (years)*	*Mean age (years)*	*Patients* < *1 year included (yes/no)*	*Minimum n. of patients* < *1 y (available data*^b^*)*	*Maximum n. of patients* < *1 y (available data*^b^*)*	*Number of patients* < *1 year*
*Total*	1663	908 (54.6%)	755 (45.4%)	0 to 24		4.2^a^		5/1663 (0.3%)	41/1663 (2.4%)	5 to 41 out of 1663 (0.3 to 2.4%)
*Connolly AM *et al*. 1994 *[9]	73	41 (56.2%)	32 (43.8%)	1 to 21	N.A.^a^	5.37	No	/	/	/
*Evald L. *et al*. 2020I *[26}	3	2 (66.6%)	1 (33.4%)	3 to 15	4.3	4.6	No	/	/	/
*García-Iñiguez *et al*. 2019 *[27]	9	5 (55.5%)	4 (44.5%)	3 to 12	6.5	7.6	No	/	/	/
*Garone G. *et al*. 2019* [6]	509	273 (53.6%)	236 (46.4%)	1 to 18	4.4	5.8	No	/	/	/
*Gieron-Korthals *et al*. 1994* [28}	40	14 (35%)	26 (65%)	0 to 18	N.A	6.6	Yes	1	24	Not specified
*Harai T. *et al*. 2010I* [29]	1	1 (100%)	0 (0%)	7	7	7	No	/	/	/
*Hata A. *et al*. 2008* [30]	2	1 (50%)	1 (50%)	1.3	1.3	1.3	No	/	/	/
*Hennes E. *et al*. 2012* [31]	11	6 (54.5%)	5 (45.5%)	3 to 14	6.7	6.9	No	/	/	/
*Kirik S. *et al*. 2021* [5]	39	21 (53.8%)	18 (46.2%)	1.5 to 16	N.A	6.2	No	/	/	/
*Kornreich L. *et al*. 2016 *[32]	9	5 (55.5%)	4 (44.5%)	3 to 13	5.8	6.8	No	/	/	/
*Lancella L. *et al*. 2017* [33]	124	74 (59.7%)	50 (40.3%)	1 to 18	N.A	5.3	No	/	/	/
*Luetje M. *et al*. 2019* [34]	141	79 (56%)	62 (44%)	15 months to 18 years	N.A	5.3	No	/	/	/
*Martínez-G M. *et al*. 2006* [35]	39	23 (59%)	16 (41%)	1.5 to 5	N.A	4.3	No	/	/	/
*Muthusamy K. *et al*. 2019* [36]	22	10 (45.4%)	12 (54.6%)	10 months to 3.1 years	N.A	1.75	Yes	1	14	Not specified
*Nussinovitch M. *et al*. 2003* [37]	39	21 (53.8%)	18 (46.2%)	1 to 24	N.A	4.8	No	/	/	/
*Rudloe T. *et al*. 2015* [38]	364	196 (53.8%)	168 (46.2%)	1.7 to 5.5	2.8	N.A	No	/	/	/
*Segal E. *et al*. 2019* [39]	58	35 (60.3%)	23 (39.7%)	1 to 14	N.A	4.9	No	/	/	/
*Thakkar K. *et al*. 2016* [7]	120	66 (55%)	54 (45%)	6 months to 18 years	N.A	4.5	Yes	3	3	3
*Van der Maas *et al*. 2009 *[14]	45	25 (55.5%)	20 (44.5%)	1.4 to 12.9	2.8	3.7	No	/	/	/
*Yildirim M. *et al*. 2020 *[15]	15	10 (66.6%)	5 (33.4%)	3 to 15	9.5	9.1	No	/	/	/

^a^According to available data; N.A. not available

^b^A possible range of patients < 1 year of age has been defined following the available data

Multicomponent MenB vaccine (Bexsero) leads to the production of antibodies directed against NHBA, NadA, fHbp, and PorA P1.4. The susceptibility of MenB the immune response following the vaccination depends on the antigenic similarity of the bacterial and vaccine antigens, as well as the amount of antigen expressed on the surface of the invading bacteria.

Nonetheless, the benefit-risk balance for Bexsero and other MenB vaccines has to be considered very favorable, as determined by regulatory authorities [40]. Rare adverse events are generally reported at the time of marketing of any drug or vaccine, mainly due to the small numbers of participants included in pre-marketing studies. A German pharmacovigilance study conducted between 2013 and 2016 and published in 2018 found several neurological adverse effects, mainly reported between 28 days and 11 years of age, with a significant incidence between 28 days and 23 months [23]. These included headache, febrile seizures, neuromuscular and neurological disorders. The analysis did not indicate any emerging safety signal. Rather, results were consistent with the known safety profile of the MenB vaccine. To the authors’ knowledge this is the first case of ACA following this MenB vaccine reported so far. We also report that this ACA side effect was benign with a full recovery within a month.

We also performed a narrative review of the literature of the last 50 years identifying six articles about post-vaccine ACA including six patients (Table 2) [17–21, 41]. In order to critically review the cases reported and to rigorously evaluate the causal relationship of our patient we reassessed the published reports and our case report through the World Health Organization (WHO) causality assessment criteria [42, 43]. The six published patients range from 1 year and 11 months to 26 years, with a mean latency time from vaccination to first symptoms of 6.8 days and a subsequent complete clinical recovery. Using the WHO criteria, we evaluated the reported vaccine-induced ACA cases.

**Table 2:** Cases of post-vaccine acute cerebellar ataxia

	***Vaccination***	***Type of vaccine***	***Number of patients***	***Sex***	***Age of patients (years)***	***Latency time from vaccination***	***Causality according to the author***	***WHO causality assessment criteria***	***WHO causality assessment criteria reevaluation***
*Cutroneo P. M. *et al*. 2014* [21]	Meningococcus C	Meningococcal group C conjugate vaccination	1	Female	12	1 day	Very likely	Yes	Very likely (authors followed the WHO criteria)
*Yonee K. *et al*. 2013* [41]	Human Papillomavirus	HPV-16/18 AS04-adjuvanted cervical cancer vaccine	1	Female	12.5	12 days	Very likely	Not declared	Very likely
*Deisenhammer F. *et al*. 1994 *[18]	Hepatitis B	Booster bivalent HBV vaccination	1	Female	26	10 days	Probable	Previous publications	Probable
*Saito H. *et al*. 1989* [17]	Influenza, The vaccine contained	Hemagglutinin particles equivalent to influenza A/Kumamoto/37/79/(H1N1) Russian strain 250 CCA/ml, influenza A/Niigata/102/81(H3N2) 300 CCA/ml, and influenza B/Singapore /222/79 150 CCA/ml	1	Female	5	8 days	Probable	Previous publications	Probable
*Katafuchi Y. *et al*. 1989* [19]	DTP	N.A	1	Female	1 y e 11 months	5 h	Very likely	Previous publications	Not applicable (Japanese article, abstract in English)
*Sunaga Y. *Et al*. 1995* [20]	Varicella	N.A	1	Male	2	10 days	Possible	Previous publications	Possible

In our case, we describe that cerebellar symptoms started within 24 h from the vaccination booster, with no evidence of other possible causes. Overall, considering the hypothesis of an immune mediated reaction to vaccine components and after exclusion of other causes, vaccination can be considered as a possible trigger of ACA, although it cannot be proved, and it cannot be classified as certain cause. Despite the temporal association with the vaccination, we consider an unknown infective trigger still possible, as it the the most frequently identified cause at our age presentation [37]. In their study Kirik et al. could not identify a certain cause in almost 90% of patients [5]. According to the WHO criteria [42], our ACA case can be classified as ‘‘probable’’ to be vaccine-associated.

Our experience and the review of literature show that when a patient with ACA after recent vaccination is evaluated a rigorous diagnostic work-up and evaluation need to be performed. Although we have raised the possibility of a causal relationship between our patient's ataxia and the vaccine, it is important to consider a range of differential diagnoses before reaching any conclusion and to be aware of the level of certainty reached. Several possible etiologies, including infectious, inflammatory, genetic, and metabolic conditions, may lead to cerebellar ataxia in children [39, 44]. In our patient, we performed a thorough clinical examination, along with laboratory and imaging studies, to exclude these other causes of ataxia. To exclude influenza A/B and other respiratory viruses as a possible cause of the patient's ACA, we performed PCR testing on blood, nasopharyngeal aspirate and cerebrospinal fluid samples, which did not detect any evidence of a respiratory virus infection. Moreover, even if most cases of ACA in children are idiopathic or associated with virus infections, a subset of cases may be caused by intoxication with various agents [45]. Indeed, the cerebellum is particularly vulnerable to toxic insults, and the cerebellar cortex and Purkinje neurons are often the main targets of toxic damage. Alcohol is the most common cause of toxic cerebellar lesions in humans, but a variety of drugs and environmental toxins can also induce cerebellar deficits. Anticonvulsants, antineoplastics, lithium salts, and calcineurin inhibitors are examples of drugs that can cause cerebellar ataxia [46]. Drug abuse and addiction, such as cocaine, heroin, and phencyclidine, can also result in cerebellar damage. Exposure to environmental toxins, such as mercury, lead, manganese, and toluene/benzene derivatives, can also lead to cerebellar dysfunction [45].

Although the prevalence of cerebellar lesions related to intoxication and poisoning are still unknown in many cases, clinicians should be aware of the agents that can cause cerebellar deficits. It is important for clinicians to consider toxic exposure in children with cerebellar ataxia, especially when the patient's clinical status suggests life-threatening intoxication. While the lack of investigation into intoxication causes represent a limitation of our case report, it is important to note that given the history and anamnesis provided by the parents we did not pursue investigation into potential intoxication causes as part of our diagnostic workup. However, we acknowledge that the cerebellum is particularly vulnerable to intoxication, and that ACA related to toxin exposure is not rare in clinical practice, even in pediatrics.

Though the role of MenB vaccination in development of ACA has to be clarified, this case may be unique in detailing an ACA presentation under 12 months of age. Post-marketing surveillance is confirmed to be an essential tool for collecting all suspected reports to better identify possible correlations, and further research is then needed to clarify the complex pathogenesis of this disorder and its eventual link with vaccinations. It is important to note that vaccines are a crucial tool for preventing many infectious diseases, and that the administration of vaccines is considered safe and well-tolerated.

Our experience highlights that a rigorous scientific approach is needed to establish the cause-effect relationship in suspected vaccination reactions. Our review shows that ACA under 1 year of age is a rare event, and it provides clear reassurance regarding the rarity and self-limited benign evolution of ACA occurring after vaccination, although the causality between the few ACA cases reported and vaccination remain unproved.

## Data Availability

Data and written consents are available from the corresponding author upon reasonable request.

## Abbreviations

CNS: Central nervous system
ACA: Acute cerebellar ataxia
MenB: Meningococcal group B
WHO: World Health Organization
